# Changing patterns of adult asthma incidence: results from the National Health Insurance Service–National Sample Cohort (NHIS-NSC) database in Korea

**DOI:** 10.1038/s41598-018-33316-y

**Published:** 2018-10-09

**Authors:** Ji-Yeon Shin, Kyoung-Hee Sohn, Ji Eun Shin, Mira Park, Jiseun Lim, Jin Yong Lee, Min-Suk Yang

**Affiliations:** 1 Department of Preventive Medicine, School of Medicine, Kyungpook National University, Daegu, Korea; 2 Division of Allergy and Clinical Immunology, Department of Internal Medicine, Seoul National University College of Medicine, Seoul, Korea; 3 Department of Preventive Medicine, School of Medicine, Eulji University, Daejeon, Korea; 4 Public Health Medical Service, Boramae Medical Center, Seoul National University College of Medicine, Seoul, Korea; 5 Department of Internal Medicine, Boramae Medical Center, Seoul National University College of Medicine, Seoul, Korea

## Abstract

This study was conducted to assess the changes in the annual incidence of adult asthma in Korea where the prevalence of asthma had increased steadily in recent decades. A population-based cohort study was conducted using the National Health Insurance Service–National Sample Cohort (NHIS-NSC), which consisted of 746,816 adults aged >20 years between 2004 and 2012. Asthma was defined by two or more physician claims on the basis of a primary diagnostic code for asthma and administration of asthma medications within 1 year. The incidence rates and annual percent change were calculated, and the influence of age and sex on the incidence rates was studied. The annual asthma incidence increased from 3.63 in 2004 to 6.07 per 1,000 person-years in 2008. Since 2008, the asthma incidence did not change significantly. The asthma incidence was higher in women than in men throughout the study periods (p < 0.001) and higher in older than younger age groups (p < 0.001). The asthma incidence did not change in all ages since 2008, except for the 20 s who showed a steady increase. The incidence of asthma in adults reached plateau in Korea, which is consistent with the results from studies in other countries.

## Introduction

Asthma is the most common chronic airway disease, affecting approximately 358 million people in 2015 globally^1^. Although the age-standardised death rate largely decreased by 58.8% between 1990 and 2015, a total of 0.4 million people still died from asthma worldwide^1^. Thus, asthma was ranked 23rd as the cause of disease burden measured by disability-adjusted life years in 2015^1^. The cost for asthma management in Korea was estimated in 2004: the total costs of asthma as the sum of the direct and indirect costs was $2.04 billion, and when intangible costs were included, the total costs increased even higher, up to $4.11 billion^2^. Moreover, because asthma is often accompanied by other chronic respiratory diseases, asthma-related costs were expected to be higher^3^. However, asthma is a one of the ambulatory care-sensitive conditions in which the deterioration and admission can be prevented by effective outpatient treatment^4^. Therefore, up-to-date population data for asthma are one of the most valuable factors for policy making on national health.

The prevalence and incidence of asthma in different countries varies widely. The incidence of childhood asthma has sharply increased since the mid-1900s, especially in Western Europe. According to the International Study of Asthma and Allergies in Childhood (ISAAC) I, differences in asthma prevalence were >15-fold between English-speaking countries and other parts of globe, such as Eastern Europe and Asia in 1994 and 1995^5^. However, the disparity is narrowing owing to the increasing prevalence of asthma in low- and middle-income countries and decelerating in high-income countries^6^. For adult asthma, an international assessment of its prevalence (the European Community Respiratory Health Survey or ECRHS) was conducted between 1991 and 1994. Like ISAAC, ECRHS found a high prevalence of asthma in English-speaking countries and Western Europe, with a lower prevalence in Eastern and Southern Europe^7^. The prevalence of adult asthma has also levelled off in the past decade at least in Western Europe^8^. In Korea, the prevalence of asthma was assessed by various investigators, and the results varied widely^9–13^. Although methodological issues such as heterogeneity of asthma definition existed among studies, the increase in asthma prevalence in Korea seems to be lower or stable for the last 10 years in both children and adults than that in the previous decades^12,13^. However, unlike the studies on asthma prevalence, reports on asthma incidence have been limited^14–19^, especially in adults in Asian countries^17^. Therefore, this study aimed to investigate the incidence of adult asthma using the National Health Insurance cohort in Korea.

## Results

Table 1 shows the annual incidence rates of asthma in adults from 2004 to 2012. The incidence increased from 2004 to 2008 and then decreased from 2008 to 2012. The age-adjusted incidence rate per 1,000 PYs was 3.63 in 2004, 6.07 in 2008, and 5.35 in 2012. Trend analysis using joinpoint regression indicates that the age-adjusted asthma incidence rate significantly increased until 2008 (APC 13.8) and decreased from 2008, but it was statistically insignificant (APC −2.9). From 2004 to 2012, the average annual increase in asthma incidence was 5.1% per year (Table 2).

**Table 1:** Trends in crude and age-adjusted incidence rates of asthma in adults (per 1,000 person-years) from 2004 to 2012.

		Year								
		2004	2005	2006	2007	2008	2009	2010	2011	2012
Total	Person-years	682,642.70	659,063.10	637,373.50	638,397.60	615,712.60	603,158.10	595,444.90	587,819.90	582,266.10
	Numbers of incident cases	2,355	2,542	2,727	3,440	3,890	3,600	3,398	3,434	3,480
	Crude incidence rate	3.45	3.86	4.28	5.39	6.32	5.97	5.71	5.84	5.98
	Age-adjusted incidence rate^*^	3.63	3.96	4.3	5.28	6.07	5.67	5.35	5.35	5.38
	Age and sex adjusted incidence rate†	3.71	4.07	4.42	5.47	6.3	5.83	5.45	5.46	5.46
Men	Person-years	343,125.40	331,413.90	320,623.40	321,353.30	311,351.80	305,140.30	301,655.20	298,058.60	295,458.10
	Numbers of incident cases	924	966	1,092	1,261	1,461	1,295	1,298	1,275	1,303
	Crude incidence rate	2.69	2.91	3.41	3.92	4.69	4.24	4.3	4.28	4.41
	Age-adjusted incidence rate^*^	3.13	3.31	3.77	4.23	4.94	4.34	4.28	4.14	4.14
Women	Person-years of observation	339,517.30	327,649.10	316,750.20	317,044.40	304,360.80	298,017.80	293,789.80	289,761.30	286,808.00
	Numbers of incident cases	1,431	1,576	1,635	2,179	2,429	2,305	2,100	2,159	2,177
	Crude incidence rate	4.21	4.81	5.16	6.87	7.98	7.73	7.15	7.45	7.59
	Age-adjusted incidence rate^*^	4.53	5.09	5.37	7.03	8.04	7.65	6.93	7.09	7.08

^*^Adjusted to the 2005 Korean population census by direct standardization method.

^†^Adjusted by Poisson regression analysis.

**Table 2:** Asthma incidence rate trends with joinpoint analyses from 2004 to 2012 in adults, by sex and age groups.

	Joinpoint analyses(2004–2012)						AAPC
	Years	APC	Years	APC	Years	APC	2004–2012
Total	2004–2008	13.8^*^	2008–2012	−2.9			5.1^†^
Men	2004–2008	11.5^*^	2008–2012	−3.9^*^			3.5^†^
Women	2004–2008	15.7^*^	2008–2012	−3.2			3.2
Age-group(years)							
20–29	2004–2006	22.86	2006–2009	12.65	2009–2012	0.5	10.30^†^
30–39	2004–2008	17.79^*^	2008–2012	−3.23			6.30^†^
40–49	2004–2008	13.76^*^	2008–2012	−4.25			4.40^†^
50–59	2004–2008	14.52^*^	2008–2012	−4.95^*^			4.30^†^
60–69	2004–2008	12.37^*^	2008–2012	−3.99			3.9
≥70	2004–2012	3.06^*^					3.10^†^

Abbreviations: APC, annual percent change; AAPC, average annual percent change.

Joinpoint analyses with up to 2 joinpoints yielding up to 3 trend segments (Trends 1–3) were based on rates per 1,000 persons and were age-adjusted to the 2005 Korean standard population.

^*^The APC is statistically significantly different from zero (2-sided t-test; P < 0.05).

^†^The AAPC is statistically significantly different from zero (2-sided Z test; P < 0.05).

The asthma incidence was higher in women than in men from 2004 to 2012 (Table 1; Supplementary Fig. 1). The increasing trends from 2004 to 2008 were observed in both sexes (APC 11.5 in men and 15.7 in women), but decreasing trends from 2008 to 2012 were significant only in men (Table 2).

**Figure 1:**
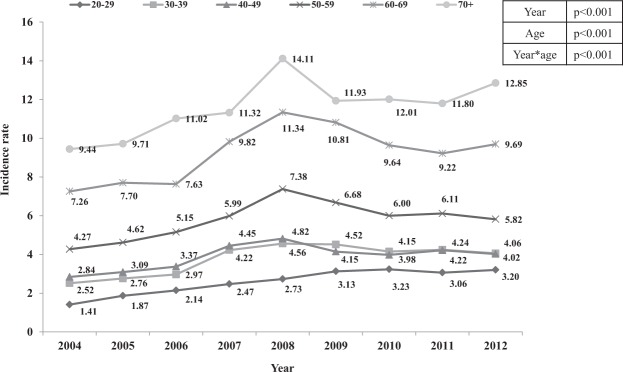
Trends of asthma incidence rates in adults (per 1000 person-years) from 2004 to 2012, by age group.

Incidence rates increased with age, and this difference increased over the calendar time. Participants aged ≥70 years showed the highest asthma incidence during 2004–2012 (Fig. 1). However, the average annual percent change during 2004–2012 was highest in those aged 20–29 years (10.3% annual increase) and decreased as the age increased, which was lowest in those aged ≥70 years (3.1% of annual increase) (Table 2).

There were no definite differences in the asthma incidence according to five income groups, although statistical significance was found (Supplementary Fig. 2).

## Discussion

The asthma incidence in Korean adults, obtained from a large longitudinal cohort, increased until 2008 and has remained stable since then. Age-adjusted asthma incidence ranged from 3.63 to 6.07 per 1,000 PYs during the study period and has been approximately 5.35 per 1,000 PYs for the last 3 years. The asthma incidence was higher in women than in men and has shown an increase with age during the study period; the asthma incidence was highest among the population aged ≥70 years during study period. The asthma incidence has remained stable since 2008 in both sex and in all ages, except the 20 s, which showed a steady increase.

The incidence of asthma in Korean adults was 3.63 to 6.07 per 1,000 person-years, ranging from 3.13–4.94 and 4.53–8.04 per 1,000 person-years in males and females, respectively. A meta-analysis of 17 studies on the incidence of asthma reported that the incidence of asthma in adults was 3.6 (95% CI 2.7–5.0) and 4.6 (95% CI 3.4–6.1) per 1,000 person-years in males and females respectively, which was slightly different from the values obtained from our study^18^. These differences could be due to the prevalence of asthma in the study year, the region in which the study was conducted and differences in the study population and the case definitions of asthma. In most westernized countries, the prevalence of asthma has increased in the 1980s and 1990s^20^, however, it had been reported that the growth of the prevalence of asthma had slowed down and even decreased since then^19,21,22^. The same trend in the changes of the prevalence of asthma had also reported in Korea from 2006 to 2010^12,13^. The most commonly used sources for studying asthma incidence are prospective cohorts to estimate the onset of a specific disease, cross-sectional data such as general population based survey data, and general population based secondary databases such as insurance claim database. The operational definitions of asthma can vary depending on the data used, the settings under investigation, and the outcome to be reported. The most precise incidence can be obtained from prospective cohort because periodic measurement of lung function and specific diagnostic tests such as bronchial provocation tests can be used. However, conducting a prospective cohort requires tremendous costs and efforts. When specific diagnostic tests cannot be performed, operational definition of asthma should be used. When using survey data, questionnaires such as “have you had asthma before?”, “have you ever had wheezing before?” or “have you ever diagnosed with asthma by a doctor?” are used to define asthma patients. When using secondary database, the diagnostic codes related to asthma, asthma related drug prescription, asthma-related tests and the combination of them can be used to define asthma^14–18^. Entering diagnostic codes, prescribing related drugs, and prescribing related tests may vary depending on the insurance system or medical environment in which the study is conducted, so it is not easy to apply universally uniform standards for operational definition of asthma. In this study, asthma cases were defined as those who were prescribed asthma medications and asthma diagnostic codes at the same time twice or more within one year, which had already been used in other studies using the same database^23^. In a similar study conducted in another countries used different operational definition of asthma according to the study setting such as a new case of asthma reported by a physician, more than three visits to the outpatient department with an asthma diagnostic codes, or at least 2 asthma physician visits within 2 consecutive years and/or at least 1 asthma hospitalization^15,17,19^. Thus, it is difficult to directly compare the results of this study with those of other studies. However, the age and gender distribution of the overall incidence of asthma from this study was not different from those from previous studies from other countries^18^.

The incidence of asthma in Korean adults had increased until 2008 and had stayed stable at about 5.35 per 1,000 since 2010. Only few researchers reported the trend of the incidence of asthma over time. In a study using a database derived from 422 primary care practices in the UK between 2001 and 2005, the incidence of asthma which was defined as the presence of a diagnostic code for asthma, in general population decreased from 6.9 (95% CI, 6.8–7.0) to 5.2 (95% CI 5.1–5.3) per 1,000 person-year (PY) during the study period^16^. The incidence of asthma decreased from 7.3 to 5.6 (−23.2% change) and from 6.5 to 4.8 (−25.8% change) per 1,000 PY in females and males, respectively. The decrease in the incidence of asthma in adults was more rapid in the elderly than the younger adults^16^. In the UK, the incidence and prevalence of asthma had increased before the 1990’s and declined since then in children and adolescents. This study confirmed that such decrease in the incidence of asthma was ongoing. Other studies also had shown the stabilization of the incidence of asthma. The incidence of adult asthma in Taiwan gradually decreased from 5.6 to 3.5 per 1,000 between 2000 and 2011 estimated from national insurance claims database^17^. The incidence of asthma was higher and decreased more rapidly in elderly population. They speculated that the decrease in the incidence of asthma was because of the success of policy against environmental pollution in Taiwan. These findings suggest that the decrease in the incidence of asthma is not a unique characteristic of our study but a common feature around the world. In Asia, this change appears earlier in regions with high prevalence of asthma^24^.

To estimate the reason of the change in the incidence of asthma, change in obesity and smoking rate which are known risk factors for asthma development should be assessed. According to the results of the National Health and Nutrition Examination Survey in 2013, the rate of obesity among Korean males increased and that among females did not changed a lot during our study period. Thus, the change in the rate of obesity could not explain the change in the incidence of asthma. Smoking rate of Korean males fell sharply until 2007 and remained around 45% and that of Korean females remained unchanged during our study period. Thus, changes in smoking rates could explain at least partly the changes in the incidence of asthma^25^. On the other hand, the basic plan for improving the air quality in the Seoul metropolitan area was established in Korea since 2005^26^. As a result, pollutants such as fine dust, sulfur oxides, and particulate matter decreased largely since then. This may have had a direct impact on changes in the incidence of asthma. However, considering that the air quality had already been improved for a long time and the incidence of asthma had decreased since the 1990s in the UK, multiple risk factors other than air quality can influence the changes in the incidence of asthma. The stabilization of asthma incidence may be interpreted at least partially that the diagnosis of asthma became earlier than before considering a steady increase in incidence in the 20 s in addition to external factors such as air pollution.

An unexpectedly surprising result was the rapid increase in asthma incidence from 2004 to 2008. The age- and sex-adjusted incidence rate of asthma increased about 70% during a 5-year period (from 3.71 per 1,000 in 2004 to 6.3 per 1,000 in 2008) and the annual increase rate was about 0.5 case per 1,000. It was quite huge considering that the annual increase in asthma incidence in the U.S. from 1980 through 1996 was 0.2 per 1,000^27^. There can be few hypotheses for this phenomenon. First, as mentioned above, the operational definition of asthma from this study would be looser than the other studies. Especially, the 2-years’ washout period would be too short that asthmatic patients in remission can be counted as new incident cases. Second, an increased awareness on asthma for both doctors and patients would lead to the increased diagnosis rate for asthma. There were social campaigns for the awareness for asthma during the early 2000s including new healthcare plans for managing allergic diseases in the community such as asthma-friendly schools and the establishment of Korean Asthma Allergy Foundation. In addition, considering that the introduction of inhaled corticosteroid and long-acting β_2_ agonist combinations to Korea was in 2000 and that of leukotriene receptor antagonist was in 2001, it would be also possible that aggressive advertising by pharmaceutical companies had led to higher asthma diagnosis for those who had asthma but had not diagnosed and treated well. Change in the healthcare system can explain such a phenomenon. However, there was no change in the healthcare system and no financial incentives for the physicians who cared for asthmatics in Korea during that period.

The strength of this study was that we could track the asthma incidence in a very large cohort that can represent the entire Korean population. There was a report that the incidence of childhood asthma did not increase anymore^28^, and the incidence of adult asthma also seemed to reach the plateau when the results of various studies were referred. However, because of the methodological differences between epidemiological studies as described above, direct comparisons were difficult. In this study, we could show by following up a single population longitudinally, that there was a real inflection point in the incidence of adult asthma.

The limitation of this study is that the incidence of asthma may be overestimated because there can be asthmatic patients those who remain asymptomatic without medication and who have symptoms but do not seek medical care for years. To reduce such risks, we set 2-years’ washout period before analysis. However, 2 years’ washout period would not be enough to eliminate prevalent asthmatics who do not use medical sources. However, as we focused on the trend of incidence rather than the incidence itself, we wanted to follow as long period as possible within a limited time period of the longitudinal cohort. The diagnosis of asthma may be inaccurate because the claim data was used and the asthma patient was defined using only the diagnostic code and drug prescription code. It was also possible that the incidence is underestimated because it is not included in asthma patients who have not used medical care.

In conclusion, this study estimated the trends in the incidence of asthma for 9 years using a large longitudinal cohort from the claims database. During the study period, the incidence of asthma in Korea ranged from 3.63 to 6.07 per 1,000 person-years, with a higher incidence in women and the elderly. The incidence of adult asthma in Korea no longer increased from 2008, which directly showed the plateauing of the asthma incidence. Further research is needed to determine the cause of this phenomenon.

## Methods

### Database and study cohort

We conducted a retrospective population-based cohort study using the National Health Insurance Service–National Sample Cohort (NHIS-NSC) 2002–2013^29^. The data were produced by the Korea National Health Insurance Service (KNHIS) using a systematic sampling method to generate a representative sample from all 46,605,433 Korean residents in 2002, comprising a total of 1,025,340 nationally representative randomly selected subjects or 2.2% of the entire population in 2002 who were followed up for 11 years until 2013. The representativeness of the data had been presented elsewhere^29^. The data that support the findings of this study are available from the Korea National Health Insurance Service but restrictions apply to the availability of these data, which were used under license for the current study, and so are not publicly available. Data are however available from the Korea National Health Insurance Service upon reasonable request and with the appropriate review process. This database includes all medical claims filed from January 2002 to December 2013. More details of the cohort are described elsewhere^29^.

There were 746,816 adults (aged 20 or over), eligible participants for our study among total 1,025,340 cohort participants in 2002. In order to yield the incidence of adult asthma from 2004 to 2013, we set up two year wash-out period. We excluded the participants who have diagnosed with asthma (ICD 10 code J45 or J46) from 2002 to 2003. Finally, 686,182 participants were included as our study population and we followed them to detect their asthma onset from January 1, 2004, to December 31, 2013. All participants were monitored from the study entry to the end of the study period unless their eligibility was disqualified due to death or emigration.

### Asthma case definition

We defined the incident cases of asthma by reviewing previous studies that operationally defined asthma cases using healthcare claim data^16,17,30,31^. As a result, if individuals had at least two or more physician claims with a primary (first listed) diagnosis of asthma (ICD 10 code J45 or J46) with at least one of the prescription of following asthma-related medications (inhaled corticosteroids (ICSs), ICSs combined with long-acting β2-agonists (LABAs), oral leukotriene antagonists (LTRAs), short-acting β 2-agonists (SABAs), systemic LABAs, xanthine derivatives, and systemic corticosteroids) for the duration of 1 year interval and had no asthma diagnoses in the claims data sets of the calendar year before their first asthma diagnosis, they were defined as incident asthma cases in the specific year.

### Statistical analysis

To calculate the follow-up time, the date of the first medical visit meeting the asthma definition and date of disqualification due to death or emigration, whichever occurred first, were obtained. The study period for assessing annual asthma incidence was from 2004 to 2012.

Person-years (PYs) were calculated for each calendar year, age (based on the first day of each year), and sex. Incidence rates were determined by dividing the total number of newly diagnosed asthma cases by the total number of PYs accumulated. Crude rates, age-, sex-, and income level-specific incidence rates per 1,000 PYs were calculated. Age-adjusted incidence rates were calculated using direct standardized methods based on the 2005 Korean population census as the standard population. Age was categorized into 20–29, 30–39, 40–49, 50–59, 60–69, and ≥70 years. Income level was categorized into quintiles based on household National Health Insurance premium.

Poisson regression analysis was used to calculate sex- and age (categorical)-adjusted incidence rates and to test the difference in incidence trends according to age, sex, and income groups (i.e., interaction with the calendar year). All statistical analyses were performed using the SAS software package version 9.4 (SAS Institute, Cary, NC, USA).

Trends in incidence over time were estimated using Joinpoint Regression Software^32,33^. We analyzed the trend of each line segment using the annual percent change (APC) and the overall trend for the whole study period (2004–2012) using the average APC (AAPC). Briefly, changes in the annual age-adjusted incidence rates of asthma were examined by calculating the APC over a time period as (exp(b)–1) ×100, where b is the regression slope of the log age-adjusted rate for a given calendar year^34^. A maximum of two joinpoints was allowed for each analysis. The AAPC was estimated as a weighted geometric average of APCs, with the weights equal to the length of each line segment during the prespecified, fixed interval^35^. The t-test and Z test were used to determine whether APC and AAPC were statistically different from zero, respectively. All statistical tests were two-sided. In describing trends, the terms increase or decrease were used when the slope (APC or AAPC) of the trend was statistically significant (two-sided P < 0.05). For statistically insignificant trends, terms such as stable, insignificant increase, and insignificant decrease were used^36^.

This study was approved by the Institutional Review Board of Eulji University, Korea (approval no. EUIRB2017–35). All methods were performed in accordance with the relevant guidelines and regulations approved by the Institutional Review Board of Eulji University, Korea. Informed consent was waived because all analysis used anonymous data.

## Electronic supplementary material

Supplementary figures
