# Supplementary material for: Changing patterns of adult asthma incidence: results from the National Health Insurance Service–National Sample Cohort (NHIS-NSC) database in Korea
Source: Sci Rep. 2018 Oct 9;8:15052. doi: 10.1038/s41598-018-33316-y (PMC6177405; doi:10.1038/s41598-018-33316-y)
Supplement: Supplementary file 1 — Supplementary figures [file 41598_2018_33316_MOESM1_ESM.pdf]

Changing patterns of adult asthma incidence: results from the National Health Insurance Service–National Sample Cohort (NHIS-NSC) database in Korea

Ji-Yeon Shin, MD, PhD[1], Kyoung-Hee Sohn, MD[2], Ji Eun Shin, PhD[3], Mira Park, PhD[3], Jiseun Lim, MD, PhD[3], Jin Yong Lee, MD, PhD[4,*], Min-Suk Yang, MD, PhD[2,5,*]

[1]Department of Preventive Medicine, School of Medicine, Kyungpook National University, Daegu, Korea,

[2]Division of Allergy and Clinical Immunology, Department of Internal Medicine, Seoul National University College of Medicine, Seoul, Korea,

[3]Department of Preventive Medicine, School of Medicine, Eulji University, Daejeon, Korea,

[4]Public Health Medical Service, Boramae Medical Center, Seoul National University College of Medicine, Seoul, Korea,

[5]Department of Internal Medicine, Boramae Medical Center, Seoul National University College of Medicine, Seoul, Korea

Supplementary Figure 1. Trends of age-adjusted incidence rates of asthma in adults (per 1000 person-years) from 2004 to 2012, by sex

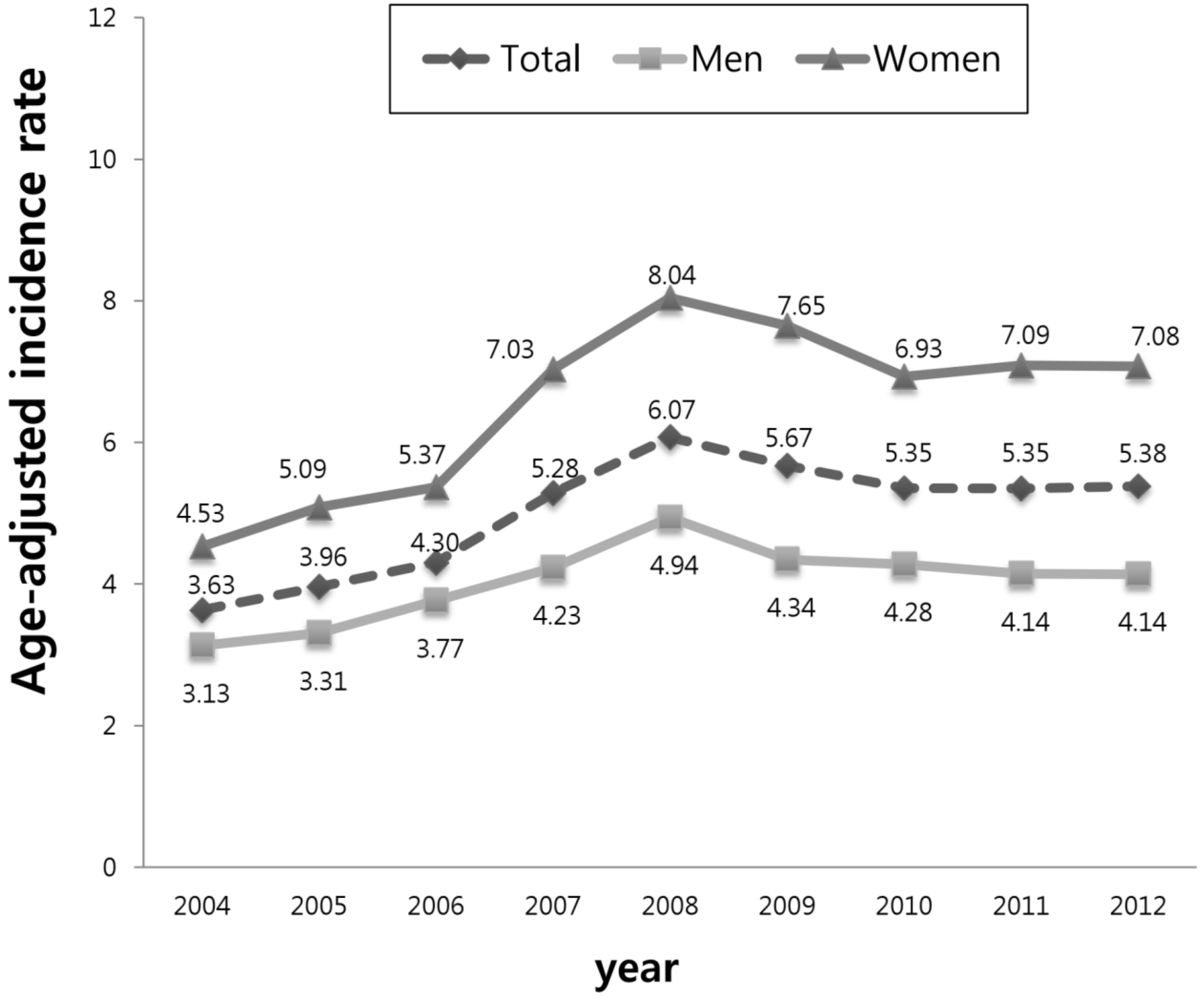

Year	p<0.001
Sex	p<0.001
Year*sex	p<0.001

Supplementary Figure 2. Trends in age- and sex-adjusted incidence rates of asthma in adults (per 1000 Person-Years) from 2004 to 2012, by income level (5 groups)

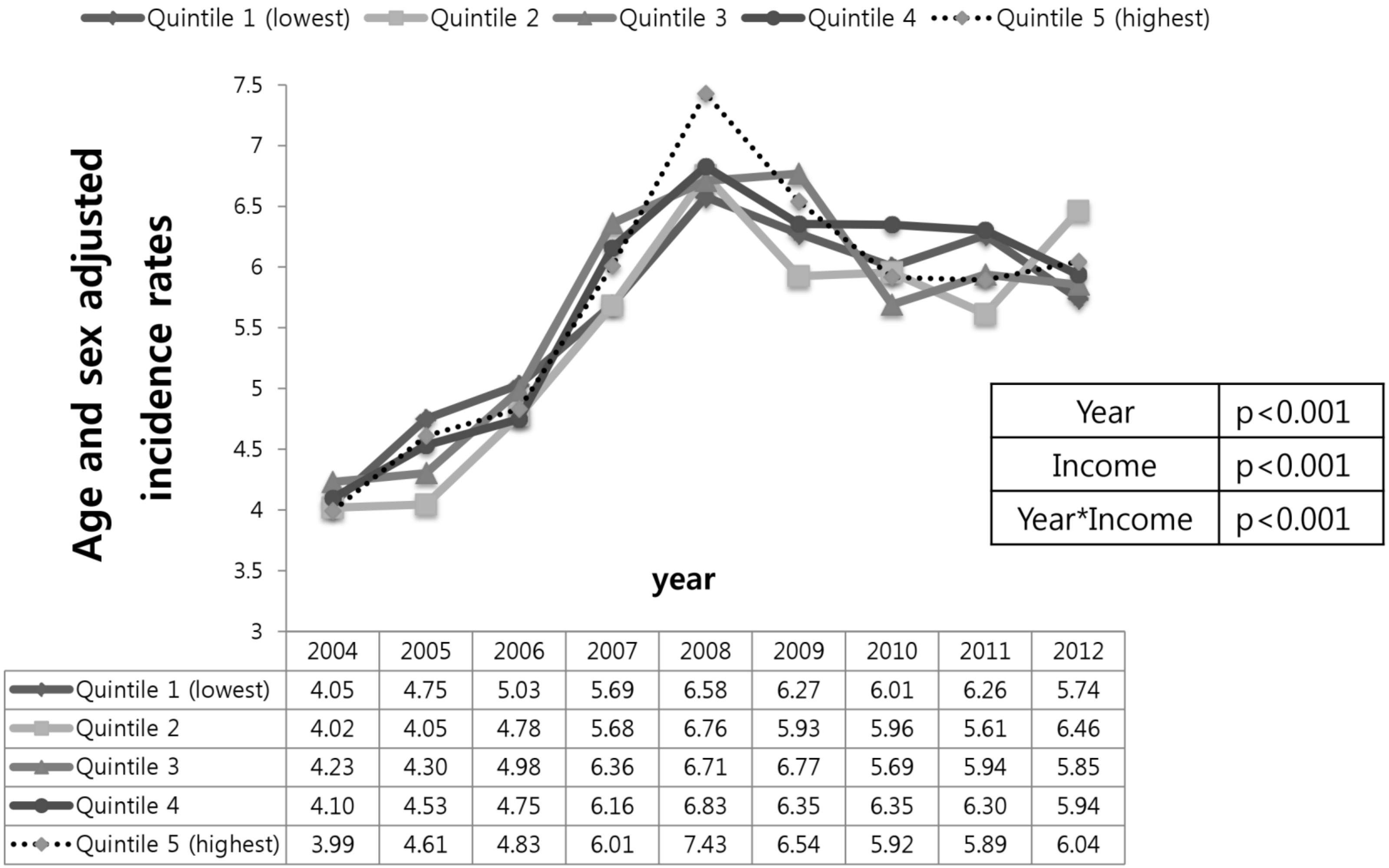

	2004	2005	2006	2007	2008	2009	2010	2011	2012
Quintile 1 (lowest)	4.05	4.75	5.03	5.69	6.58	6.27	6.01	6.26	5.74
Quintile 2	4.02	4.05	4.78	5.68	6.76	5.93	5.96	5.61	6.46
Quintile 3	4.23	4.30	4.98	6.36	6.71	6.77	5.69	5.94	5.85
Quintile 4	4.10	4.53	4.75	6.16	6.83	6.35	6.35	6.30	5.94
Quintile 5 (highest)	3.99	4.61	4.83	6.01	7.43	6.54	5.92	5.89	6.04

Year	$p<0.001$
Income	$p<0.001$
Year*Income	$p<0.001$
